# Mothers’ Employment and Exclusive Breastfeeding Practices: A Brief Report from Jerusalem Governorate

**DOI:** 10.3390/ijerph20032066

**Published:** 2023-01-23

**Authors:** Saif Amer, Elham Kateeb

**Affiliations:** 1 Horizon Academy, Al-Nayzak, Ramallah P627, Palestine; 2 Oral Health Research and Promotion Unit, Al-Quds University, Jerusalem 51000, Palestine

**Keywords:** exclusive breastfeeding, workplace policies, employment, working women

## Abstract

The World Health Organization (WHO) recommends that women exclusively breastfeed for the first six months and continue breastfeeding until two years of age. However, breastfeeding is declining, especially in developing countries. This study aims to describe breastfeeding habits and demographic factors influencing these practices in Jerusalem Governorate. Self-reporting questionnaires were sent to 481 mothers of preschoolers asking about the type of feeding used with their children, breastfeeding exclusively, bottle feeding, and a combination of both. Data were also collected about the duration of breastfeeding to classify women into those who adhered to the World Health Organization (WHO) recommendations and those who did not. We received 471 complete questionnaires. Two hundred and five mothers exclusively breastfed their children for 6 months or more (44.1%). Almost 11% (*n* = 52) used bottle feeding exclusively, and 44.2% (*n* = 208) combined both breastfeeding and bottle feeding. Having a full-time job increased the chance of not breastfeeding children (π^2^ = 9.2, *p* = 0.002), and being a stay-at-mother increased the chance of exclusive breastfeeding (π^2^ = 4.4, *p* = 0.044). In the final model, having a preterm baby and being a stay-at-home mother increased the odds of exclusively breastfeeding by 3.6 and 2.3, respectively. On the other hand, having a full-time job decreased the odds by 0.3. A mother’s full-time employment was a determinant factor in abandoning exclusive breastfeeding before 6 months. Policies, regulations, and laws supporting the promotion of exclusive breastfeeding practices until 6 months in mothers as recommended by the WHO should be reinforced.

## 1. Introduction

The World Health Organization (WHO) and United Nations International Children’s Emergency Fund (UNICEF) recommend exclusively breastfeeding infants during the first six months of life and adding complementary foods to breast milk gradually at least until the age of two years [1]. However, with changes in lifestyle and aggressive formula feeding marketing campaigns, exclusive breastfeeding is declining, especially in developing countries [2]. Globally, the prevalence of exclusive breastfeeding until the age of six months is 37% [3]. A systematic review identified several factors influencing breastfeeding practices at multiple levels: structural (socio-cultural and market factors), individual (mother related and infant related), and setting (health systems and services, family and community, and workplace and mother’s employment) [2]. The literature has identified mothers’ employment status without adequate support from the surrounding environment as a barrier to breastfeeding [4,5,6]. Issues related to work policies are major influencers on mothers’ decision to initiate breastfeeding or wean their babies sooner [7]. In 2019, 47.1% of women globally participated in the labor force [8]. Therefore, it is crucial to assess the relationship between mothers’ employment status and their feeding practices in a particular community to identify barriers and advocate for public and work-specific policies that enforce mothers’ choice to optimally breastfeed.

In Palestine, as of 2021, 23% of Palestinian women aged 25–49 were employed [9], and data from Labor Force Survey 2019 showed that the percentage of households headed by women in Palestine was 11% (12% in the West Bank and 9% in Gaza Strip) [10]. Economic and political challenges in the Palestinian community made work a necessity for some women for their families to survive.

This brief report is a secondary data analysis that aims to explore the relationship between a mother’s employment status and her breastfeeding practice. This piece of information is important to assess the need to advocate for better workplace policies that promote breastfeeding.

## 2. Materials and Methods

Mothers in this analysis were recruited from Jerusalem Governorate in areas located outside the Separating Wall. The Separating Wall was built in 2002 by the Israeli side, isolating Jerusalem’s urban center from surrounding Palestinian villages and towns. These areas were dependent on Jerusalem as their urban center for health, education, recreational activities, and social services [11]. These villages and towns, which are considered mainly rural, constituted the newly established Palestinian Authority (PA) Jerusalem Governorate, which is considered the weakest in healthcare infrastructure among all PA governorates [12].

Data in this brief report were extracted from a cross-sectional study that was designed to assess feeding practices and early childhood caries among preschoolers in the Jerusalem Governorate in the period between June 2019 and January 2020. Although this analysis was not among the aims of the original study, the authors found that exploring the relationship between a mother’s employment status and feeding practices is important for the oral and general health of the children in this sample.

**Figure 1:**
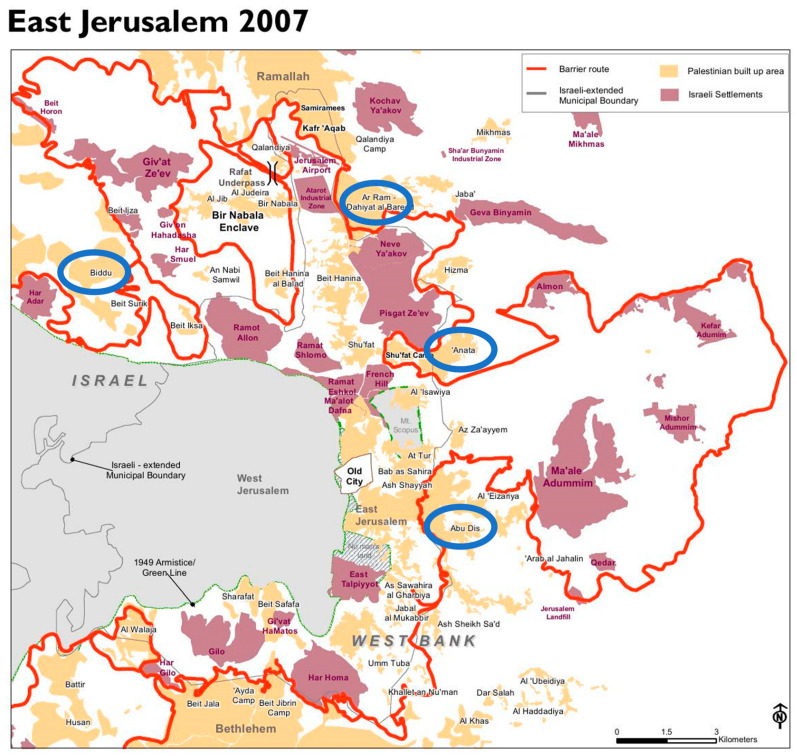
Map of the sampled schools distributed in the different areas of PA Jerusalem Governorate.

The sampling frame of this study included mothers of children registered in preschools in the PA Ministry of Education (MOE) in Jerusalem Governorate and located outside the Separating Wall (*n* = 50 schools with 4122 children). Fifteen schools (*n* = 15 schools with 1892 children) out of fifty met the study inclusion criteria. A random sample of four preschools out of fifteen were selected and stratified based on the different geographic areas in Jerusalem Governate (North, Northeast, East, and Southeast; Figure 1).

Based on sample size calculation, a minimum of 352 subjects were needed for this study. This number was based on a 5% margin error, 95% confidence level, and a target population of *n* = 4122.

Inclusion criteria for the schools were: preschools (1) located in Jerusalem Governorates and situated outside the Separating Wall, (2) registered with the Palestinian Ministry of Education, (3) including both genders, and (4) that had at least 100 children enrolled in the 2019/2020 academic year.

A self-administered questionnaire written in Arabic and adapted from previous studies [13,14] was pilot-tested on a sample of fifteen mothers in a preschool in Bethlehem Governorate. Modifications were added to make the questionnaire more readable, meaningful, and culturally appropriate. Questions in the current analysis included information on feeding practices, and demographic and socioeconomic information.

The questionnaire was sent to their home for the mothers to answer and then collected by the school administration the day before the research team’s visit to the preschools.

The socio-economic status was determined according to the mother’s level of education and family income. Information regarding the level of education was assessed using a five-point scale ranging from less than eighth-grade education to four years of college or more. The mother’s employment status was categorized as full-time job, part-time job, student, or stay-at-home. The monthly household income was given in new shekels (NIS) and ranged on a six-point scale from (1) less than 1000 NIS (USD 285) to (6) 4000 NIS (USD1700) or more. The current residence of the family was assessed as city, village, or camp.

Questions about feeding habits covered the main method of feeding during infancy, breastfeeding duration (naturally or expressed) if the mother used to breastfeed, and bottle-feeding duration using formula milk or other types of non-breast milk. 

Al-Quds University Human Subject Research Ethics Committee (74/REC/2019) reviewed all aspects of the study. Consent forms were attached to the cover letter of the questionnaire. The data pertaining to the subjects were entered into the database anonymously with a numerical code only.

Descriptive statistics were generated for the main study variables. Bivariate, and multivariable logistic regression using SPSS version 22.0 (IBM Corp. Windows, Armonk, NY, USA) were used to model the dependent variable “Exclusive Breastfeeding until 6 months”. Variables that were significant in the bivariable analysis were included in the final logistic regression model. The variables “Length of pregnancy” (full-term/preterm), “Mother’s Level of education” (5-point scale), and “Mother’s Employment Status” (dummy coded into full-time, part-time, student, stay-at-Home) were also included in the final model.

The binary dependent variable “Exclusive Breastfeeding until 6 months” (Yes/No) was developed from combining the following two questions: (1) How did you breastfeed your child (breastfeeding only, bottle feeding only, or both breastfeeding and bottle) and (2) Length of natural breastfeeding (none, less than 6 months, or 6 months or more). Mothers who answered “breastfeeding only” to the first question and “6 months or more” to the second question were combined into one variable, “Exclusive Breastfeeding until at least 6 months.”

## 3. Results

Self-reported breastfeeding habits were collected from 481 mothers in the period between June 2019 and January 2020. Demographic and socioeconomic information about the mothers are presented in Table 1.

In this sample, 211 (44.8%) mothers used breastfeeding with their children, and 205 exclusively breastfed their children for 6 months or more (44.1%; Table 2 and Table 3).

Almost 11% (*n* = 52) used bottle feeding exclusively, and 44.2% (*n* = 208) combined both breastfeeding and bottle feeding (Table 3). In 72% of the current sample (*n* = 319), mothers are the ones who initiated the weaning process. 

In the bivariate analysis, the results demonstrated that having a preterm baby was associated with a lower chance to be exclusively breastfeeding until 6 months (π^2^ = 11.1, *p* = 0.001). This agrees with the literature, which reports that premature babies are more likely to be breastfed for shorter periods than full-term babies [16].

In addition, having a full-time job increased the chance of not breastfeeding children exclusively until six months (π^2^ = 9.2, *p* = 0.002), and being a stay-at-home mother increased the chance of exclusive breastfeeding (π^2^ = 4.4, *p* = 0.044).

The variable “exclusive breastfeeding until 6 months” was modelled using forward and backward logistic regression (Table 4).

After controlling for demographic and socioeconomic factors, having a preterm baby increased the odds of exclusively breastfeeding until six months by 3.7 (CI = 1.4–9.3). Although not statistically significant in the bivariable analysis, the mother’s level of education increased the odds by 0.8 (CI = 0.67–0.96).This contradicts the previous literature that assessed factors related to exclusive breastfeeding in the Arab world [17]. However, in this sample, the level of education was highly correlated with mothers working full-time jobs, which in turn decreased the odds of exclusively breastfeeding until 6 months by 0.4 (0.22–0.67).

## 4. Discussion

This brief report demonstrates that Palestinian mothers living in Jerusalem Governorate outside the Separating Wall face the same challenges as mothers in other parts of the globe in balancing their work and the optimum breastfeeding practices recommended by the WHO. Although far less than optimum, the prevalence of exclusive breastfeeding until six months in the current sample was higher than the global average of 37% [3], the Arab World Average of 36% [17], and the average of 43.8% that was reported from different studies conducted in Palestine in the period between 2013 and 2020 [18]. When compared to the 70% prevalence of exclusive breastfeeding in three refugee camps in the Nablus area in West Bank [19], our numbers fall behind. One explanation for this high rate of exclusive breastfeeding in the refugee camp is the “Breastfeeding educational and health promotion program and policy” implemented by UNRWA (United Nations Relief and Works Agency for Palestine Refugees in the Near East). This program focused on maternal education during antenatal care visits about breastfeeding and promoted a strict policy against the promotion of formula feeding. Although the previous study [19] explored some demographic factors associated with exclusive breastfeeding, it did not assess the influence of employment status on breastfeeding practices. One reason could be the high unemployment rate among refugees in the Palestinian territories [9].

Reports from developing countries rated employment status as one of the major barriers to exclusive breastfeeding [20]. In this sample, where more than 63% came from households living below the national poverty line (compared with an average of 28% living under the national poverty line in the West Bank areas [15]), mothers need to work full-time to support the household income (almost 27% in the current sample compared with the 23% national average [10]). The mother’s full-time employment was a determinant factor in abandoning exclusive breastfeeding before 6 months. This emphasizes the fact that inadequate policies in the workplace related to breastfeeding cause inequities in terms of infant nutrition and employment choices, as well as mothers’ right to combine motherhood and their professional careers.

The literature identified several strategies that can enhance breastfeeding among working women, such as early postpartum support, maternity leave policies, teleworking, flexible working hours, and access to space and time to extract human milk [6,9].

According to PA Labor Law, women workers in the Palestinian territory are entitled to a paid maternity leave of 10 weeks (70 days), of which 6 are postnatal, as well as to nursing breaks during work hours [21]. A literature review conducted in 2018 [22] demonstrated a positive relationship between maternity leave length and breastfeeding and emphasized that public health policies should ensure that all women, regardless of their economic status, should have equal access to the benefits of adequate maternity leave.

This study did not assess the mothers’ knowledge about and attitude toward breastfeeding, which are key factors in the decision to exclusively breastfeed until six months. This is a limitation of this analysis and a future direction for more exploration. Including only mothers of children in preschools might cause selection bias towards working mothers; however, preschools included in this sample are kindergartens that enroll children older than four years and are not considered daycares for working mothers. The data collected in this study were bi-directional and can only suggest that employment status may influence working mothers’ breastfeeding practices. 

## 5. Conclusions

A mother’s full-time employment was a determinant factor in abandoning exclusive breastfeeding before 6 months. According to a 2015 UNICEF report about breastfeeding in Palestine [23], initially, 96% of Palestinian mothers exclusively breastfed their infants at birth. In our report, this drops to 44% who exclusively breastfed their children until the age of six months. The practices of breastfeeding and exclusive breastfeeding until six months in this sample can be improved by designing interventions that target structural, setting, and individual factors [2]. The literature demonstrated that addressing setting factors related to workplace policies showed a good impact on more favorable breastfeeding practices [24]. In Palestine, especially in Jerusalem Governorate, where the economy and healthcare are challenging, innovative policies and interventions to modify workplaces to promote mothers’ exclusive breastfeeding until 6 months should be implemented. Policies, regulations, and laws supporting the promotion of mothers’ exclusive breastfeeding practices until 6 months as recommended by the WHO should be reinforced.

## Figures and Tables

**Table 1:** Sample characteristics.

Variable	Frequency	Valid Percent
**Gender of the Child**		
Male	250	52.3
Female	228	47.5
No answer	3	
**Length of Pregnancy**		
Preterm	35	7.5
Full-term	432	92.5
No answer	14	
**Mother’s Education Level**		
8th grade or lower	20	4.2
Lower than high school	87	18.3
Finished high school	82	17.2
Diploma—2 Years or more	66	13.9
Bachelors—4 Years or more	221	46.4
No answer	5	
**Mother’s Employment**		
Full-time	107	22.6
Part-time	21	4.4
Student	5	1.1
Stay-at-home mother	341	71.9
No answer	7	
Household Monthly Income *		
Less than USD 285	21	4.7
USD 285-570	37	8.3
USD 571-856	112	25.1
USD 857-1142	109	24.4
USD 1143- 1714	113	25.3
USD 1715 and more	54	12.1
No answer	35	
**“Do both parents work outside the house?”**		
Yes	197	44.2
No	249	55.6
No answer	35	
**Current Living Area**		
City	97	20.7
Village	367	78.3
Camp	5	1.1
No answer	12	

* In 2017, the poverty line and the deep poverty line for a reference household of five individuals (two adults and three children) were, respectively, USD 705 and USD 564 [15].

**Table 2:** Types of infant feeding practice (*n* = 471).

Type of Infant Feeding		Frequency	Valid Percent
**Valid**	Breastfeeding	211	44.8
	Bottle feeding	52	11.0
	Both breastfeeding and bottle feeding	208	44.2
	No answer	10	2.1
	Total	471	100.0

**Table 3:** Frequency of exclusive breastfeeding until 6 months and beyond (*n* = 465).

Exclusive Breastfeeding until 6 Months		Frequency	Valid Percent
**Valid**	Yes	205	44.1
	No	260	55.9
	No answer	16	3.3
	Total	465	100.0

**Table 4:** Logistic regression of the binary dependent variable “Exclusive Breast Feeding until 6 months (Yes/NO)” (*n* = 465).

Predictor Variable	Wald	df	Sig.	Exp(B)	95% C.I. for EXP(B)	
					Lower	Upper
Mother‘s FT Employment	11.308	1	0.001	0.383	0.219	0.670
Length of Pregnancy	7.452	1	0.006	3.675	1.443	9.354
Mother’s Education Level	5.865	1	0.015	0.802	0.670	0.959
Constant	2.467	1	0.116	3.941		

## Data Availability

The datasets used and analyzed in the current study are available from the corresponding author upon reasonable request.
